# Bone transport in the management of fracture-related infection: current concepts and innovations

**DOI:** 10.5194/jbji-11-191-2026

**Published:** 2026-04-01

**Authors:** Willem-Jan Metsemakers, Austin T. Fragomen, Mario Morgenstern, Steffen B. Rosslenbroich, Stephen M. Quinnan, Pablo S. Corona, Mitchell Bernstein, Kevin Tetsworth

**Affiliations:** 1 Department of Trauma Surgery, University Hospitals Leuven, Leuven, Belgium; 2 Department of Development and Regeneration, KU Leuven, University of Leuven, Leuven, Belgium; 3 Limb Lengthening & Complex Reconstruction Service, Hospital for Special Surgery, New York, NY, USA; 4 Center for Musculoskeletal Infections, Department of Orthopaedic and Trauma Surgery, University Hospital Basel, Basel, Switzerland; 5 Department of Trauma, Hand and Reconstructive Surgery, St. Mary's Hospital Osnabrueck, Osnabrueck, Germany; 6 Department of Orthopedics, St. Mary's Medical Center, West Palm Beach, Florida, USA; 7 Department of Orthopaedic Surgery, Hospital Universitario Vall d'Hebron, Universidad Autónoma de Barcelona, Barcelona, Spain; 8 Reconstructive and Septic Surgery Unit, Hospital Universitario Vall d'Hebron, Universidad Autónoma de Barcelona, Barcelona, Spain; 9 Departments of Surgery & Pediatric Surgery, McGill University Montreal, Quebec, Canada; 10 Department of Orthopaedic Surgery, Royal Brisbane and Women's Hospital, Brisbane, Queensland, Australia

## Abstract

Despite advances in musculoskeletal trauma care, segmental bone loss remains a major clinical challenge. A substantial proportion of these cases are associated with fracture-related infection (FRI), which fundamentally alters the biological environment and reconstructive strategy. In this context, FRI should be considered in any patient with a segmental bone defect, and thorough surgical debridement with acquisition of multiple deep-tissue cultures represents an essential first step in management. The presence of infection reduces the likelihood of bone consolidation and eradication of disease, emphasizing the need for strict adherence to established FRI treatment principles. Depending on the extent of bone loss, host factors, and available expertise, patients may be eligible for a range of reconstructive options. Bone transport is one of these surgical methods and involves the gradual translocation of bone segments to reconstruct defects in long bones. Based on the principles of distraction osteogenesis (DO), controlled mechanical distraction promotes predictable bone regeneration according to Ilizarov's principles. Traditionally, DO has relied on circular external fixators. Recent developments involving integrated fixation, such as bone transport over a nail, have reduced morbidity and enabled faster reconstruction. Although these techniques carry a risk of contaminating the intramedullary canal, exclusive long-term use of external fixators is not harmless. Further innovations, such as motorized telescopic intramedullary nails, have led to the gradual replacement of external fixation, making the procedure less burdensome for patients. Advances in surgical skills and technology have enabled the treatment of more complex cases, making a specialized, multidisciplinary approach essential in modern clinical care.

## 1 Introduction

Despite advances in the management of musculoskeletal trauma, segmental bone loss (or “critical-sized” defects) continues to pose significant clinical challenges even for experienced surgeons (Metsemakers et al., 2019). Importantly, a substantial proportion of these defects are related to fracture-related infection (FRI), either as a direct consequence of the initial injury or repeated surgical interventions, or as the unavoidable result of radical debridement required for infection control. The presence of FRI profoundly alters both the biological environment and the reconstructive pathway, reducing the likelihood of successful bone consolidation and increasing the risk of persistent or recurrent infection (Bezstarosti et al., 2021). The psychosocial burden and substantial financial impact on those patients affected by these conditions should not be underestimated (Garabano et al., 2025; Iliaens et al., 2021; Norris et al., 2021).

Depending on the extent of bone loss, surgeon skills, and institutional resources, a patient might be eligible for a wide range of surgical and non-surgical management options (Bezstarosti et al., 2021; Kadhim et al., 2017). For small bone defects, treatment using autologous bone grafting or bone substitutes has a long history of satisfactory clinical results. In certain cases of small defects, accepting a minor leg length discrepancy resulting from a procedure called acute shortening may be the most practical approach; alternatively, acute shortening and subsequent lengthening can be a valid option. However, for large bone defects, more invasive treatment methods are required.

**Figure 1:**
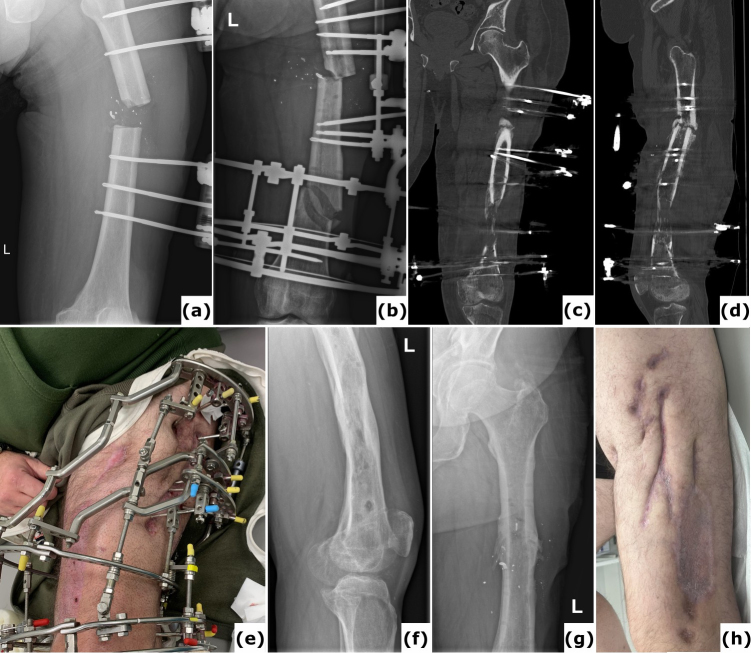
A case of polymicrobial fracture-related infection (FRI) of the femur resulting from a gunshot injury is described. A 44-year-old polytrauma patient presented with multiple injuries, including a Gustilo-Anderson type III open femur fracture of the left lower limb. **(a)** The patient was transferred after initial stabilization, external fixation of the left femur, and several surgical wound debridements. **(b)** Anteroposterior radiograph of the left femur was taken following acute shortening, distal corticotomy, and lengthening. No cultures were collected during this surgery. **(c–d)** Computed tomography (CT) images of the left femur at 6 months show a persistent unhealed fracture site along with a distal distraction site that is not fully consolidated. Due to the presence of suggestive signs of FRI (local redness, swelling, and elevated serum inflammatory markers), local debridement of the fracture site was undertaken. Cultures grew *Klebsiella pneumoniae* and *Enterococcus faecalis*, for which the patient received systemic antibiotic therapy (6 weeks). **(e)** Clinical image of a classic ring fixator. **(f–g)** Lateral and anteroposterior radiographs after removal of the external fixator at 2 years demonstrate consolidation of the fracture and the distal distraction site. Because of continued pin site infections, local redness, and elevated serum inflammatory markers, the intramedullary canal was reamed and cultures were obtained, all of which were positive for *Staphylococcus aureus*. Again, the patient received systemic antibiotic therapy (6 weeks). **(h)** Clinical images of the lower limb 1 year after removal of the external fixator show significant scarring of the left thigh from the pin tracts. Knee flexion was limited to 10°. This case shows that long-term external fixation is not harmless and can be associated with several potential complications, such as joint contracture, scarring from the pin tracts, and severe chronic infections.

Bone transport is one of these surgical methods and involves the gradual translocation of healthy bone segments to fill gaps and defects in long bones. This technique was pioneered by orthopaedic surgeon Gavriil Ilizarov in the mid-20th century and has since undergone significant refinement. The fundamental principle of bone transport relies on the body's remarkable ability to regenerate bone tissue when subjected to controlled mechanical distraction, utilizing Ilizarov's principles of distraction osteogenesis (DO) (Ilizarov, 1989). Historically, DO has been performed with the use of a circular external fixator using a low-energy corticotomy, followed by a latency period before initiating distraction through the corticotomy site. This technique, while generally considered safe and effective, is associated with several potential complications, such as nerve damage, joint contracture, scarring from the pin tracts, and (pin site) infections (Liu et al., 2023; Liu et al., 2020) (Fig. 1). Poor patient tolerance has been well documented, with one systematic review reporting that, despite low overall amputation rates, the majority of amputations were performed at the patient's request (Papakostidis et al., 2013). Recent advancements in DO feature integrated fixation approaches, encompassing planned procedures that combine both external and internal fixation methods, as well as all-internal techniques. These innovations are intended to effectively address the limitations traditionally encountered with external fixation alone.

This narrative review examines current concepts and emerging innovations in bone transport, with a deliberate focus on the central role of FRI in guiding clinical decision-making. Emphasis is placed on the need for surgeons to actively consider, diagnose, and manage FRI throughout the reconstructive process, and on how evolving bone transport techniques can be safely and effectively integrated in contemporary FRI treatment pathways.

## 2 Definitions

FRI is a well-defined clinical entity characterized by infection involving bone in the context of a healed or unhealed fracture. The internationally endorsed FRI Consensus Group has established a diagnostic framework based on two categories of criteria (Govaert et al., 2020; Metsemakers et al., 2018). Confirmatory criteria include findings such as a sinus tract communicating with the bone or implant, purulent drainage, or at least two positive deep-tissue cultures with phenotypically indistinguishable organisms. Suggestive criteria encompass clinical signs such as inflammation, radiological features indicative of infection, and elevated inflammatory markers. This standardized definition supports uniform diagnostic procedures, ensures accurate reporting in scientific publications, and informs clinical decision-making. Importantly, many segmental bone defects arise in the setting of FRI (Bezstarosti et al., 2021), and the presence of infection significantly influences both the biological environment and the reconstructive strategy. Consequently, applying a structured management pathway in accordance with the FRI Consensus guidelines is essential when evaluating treatment options such as bone transport in infected long-bone defects (Metsemakers et al., 2020b).

A critical-sized defect is a bone defect which is not expected to heal in the absence of a secondary (surgical) intervention (Bezstarosti et al., 2021). Court-Brown (Court-Brown et al., 2015) defined it as a defect involving 50 % of the cortical diameter with a minimum length of 1 cm; this was used in the Study to Prospectively Evaluate Intramedullary Nails in Tibial Fractures (SPRINT) (SPRINT Investigators et al., 2008). However, Sanders et al. later reported that, when using this definition, 47 % of the bone defects healed without additional surgery, thus indicating that these are not always critical defects (Sanders et al., 2014). At present, defects larger than 2 cm in length and with more than 50 % circumferential bone loss are considered critical defects and unlikely to heal without further intervention. This is consistent with that reported by both Haines et al. (2016) and Tetsworth et al. (2021).

Although advances have been made, there remains controversy in the orthopaedic community regarding the most effective treatment approach for critical-sized defects. A primary point of discussion concerns the indications for bone transport. Current literature generally recognizes segmental bone defects exceeding 5–6 cm as large defects, for which bone transport is considered an appropriate treatment option, particularly in the lower extremity (Sigmund et al., 2020; Tsang et al., 2024). Although this treatment approach may be appropriate for smaller defects, other strategies such as acute shortening followed by lengthening, or the Masquelet-induced membrane technique (MIMT) need to be considered.

## 3 Surgical and antimicrobial aspects

### 3.1 General considerations

**Figure 2:**
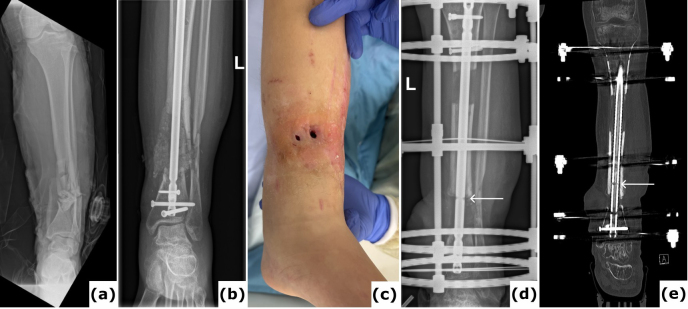
Fracture-related infection (FRI) of the tibia. An 18-year-old patient presented with a Gustilo-Anderson type III open fracture of the tibia and fibula. **(a)** Anteroposterior radiograph demonstrates a comminuted fracture of both the tibia and the fibula. **(b)** The anteroposterior radiograph indicates fixation of the fracture using an intramedullary nail. The defect was managed with MIMT. **(c)** Six months post-injury, the patient was referred with multiple fistulas, confirming FRI. **(d–e)** Anteroposterior radiograph and computed tomography (CT) images were taken after the revision procedure. Removal of the implant and thorough surgical debridement resulted in a large segmental bone defect. The images show the progression of bone transport over a 10 mm intramedullary nail. White arrows point to the off-label locking hole created during the procedure. Once bone transport is complete, the segment can be secured with a 4 mm interlocking screw and the frame can be removed, which reduces the external fixation time.

Each case should be considered a potential FRI unless evidence indicates otherwise. Therefore, the goal of every surgical revision is a judicious debridement with the removal of all implants and devitalized tissues, as well as the acquisition of deep-tissue biopsy samples for microbiology and histopathology (Moriarty et al., 2022). Bone defects can result from this surgical debridement, the infection process itself (Fig. 2), or the initial trauma. Debridement, as described by Tetsworth and Cierny (1999), is performed until bone viability is confirmed by punctate Haversian bleeding.

In addition to debridement, two key surgical considerations are achieving fracture stability and ensuring adequate soft tissue coverage. Stability of the construct in cases of segmental defects can be achieved by internal fixation, external fixation, or a combination of these techniques. These constructs are composed of three segments: a proximal, middle, and distal bony segment. The static proximal and distal segments ensure proper alignment and structural stability, while the mobile middle segment constitutes the transport segment that is incrementally advanced as a healthy section of bone across the defect site, following established principles of bone transport. These include (1) low-energy corticotomy, (2) a latency period before distraction begins, (3) regular rate and rhythm of distraction, (4) stable fixation, and (5) the active use of the limb and joints throughout treatment (Ilizarov, 1990).

Given the considerable complexity commonly observed in cases involving segmental bone defects combined with FRI, it is essential to engage a number of specialties in the decision-making process through a multidisciplinary approach (Metsemakers et al., 2020b). Accordingly, it is advisable for physicians to refer these patients to specialized centres with access to multidisciplinary teams capable of providing this comprehensive care (Metsemakers et al., 2020b).

### 3.2 Antimicrobial concepts

#### 3.2.1 Systemic antimicrobial strategies

Antimicrobials should begin with the initiation of empirical therapy, which is guided by local epidemiology and resistance patterns. Empirical antibiotics are started after deep tissue samples have been obtained during surgery, aiming to cover the most likely pathogens until specific microbiological results are available (Depypere et al., 2020a).

Once culture and sensitivity data are acquired, therapy is adjusted to a targeted regimen by selecting antibiotics with activity against the identified organism(s). The duration of targeted antibiotic therapy is not based on comparative trials but expert opinion. According to established protocols, 12 weeks of antibiotic therapy is commonplace for patients with an implant present (Depypere et al., 2020a; Moriarty et al., 2022). Cases of segmental bone defects without internal fixation, such as a ring fixator, are an exception. Here, all necrotic and infected bone is removed, and the multidisciplinary team needs to decide whether a long course of antibiotic treatment, typically 6 weeks, is necessary (Depypere et al., 2020a, b). In certain cases, it may be sufficient to administer 2 weeks of antimicrobial therapy to eradicate the residual contamination in the wound and surrounding soft tissue (Depypere et al., 2020a).

#### 3.2.2 Local antimicrobial strategies

It has become clear that local antimicrobial strategies play a pivotal role in the management of FRI by delivering high concentrations of antibiotics directly to the site, thereby maximizing delivery efficacy while minimizing systemic toxicity (Metsemakers et al., 2020a). The use of local antibiotic carriers, such as antibiotic-impregnated beads or spacers, helps to fill dead space, support the eradication of biofilm-associated bacteria, and reduce the risk of recurrence. These strategies are especially important in complex cases such as segmental bone defects, where achieving adequate systemic antibiotic levels at the infection site would be challenging or can be associated with significant side effects.

In this context, several studies have reported on the use of MIMT combined with bone transport for managing infected bone defects (Hamiti et al., 2022). In a first stage, after debridement, a polymethylmethacrylate (PMMA) cement spacer containing antibiotics is placed into the bone defect, which induces the formation of a vascularized biological membrane through a foreign-body response. In the second stage, typically after 6–8 weeks, the PMMA spacer is removed, an osteotomy performed, and bone transport initiated. The clinical outcome of bone transport through an induced membrane (BTM) is not well established, particularly regarding infection recurrence and docking site non-union rates. In a recent prospective, randomized controlled clinical study with a parallel design, the mean docking time (DT) – defined as the time to docking-site union after the completion of bone transport (months) – was significantly reduced in the BTM group (Green, 2024; Thakeb et al., 2023). There were significantly fewer postoperative complications (including docking site non-union and infection recurrence rates) in contrast to the standard BT group, with no significant differences reported for external fixation time (EFT), external fixation index, and postoperative bone and functional results between both groups. Another study described the application of this technique in a three-stage surgical protocol for reconstructing infected tibial injuries involving both bone defects and soft-tissue loss, demonstrating favourable clinical outcomes (Corona et al., 2022). Although the precise mechanism remains undetermined, these favourable results appear to be attributable to the local administration of antimicrobials rather than exclusively to the presence of the induced membrane.

More recently, newer antimicrobial strategies such as the implementation of antibacterial-coated nails (i.e. ZNN™ Bactiguard^®^ nail, EXPERT™ Tibial Nail PROtect™) could improve clinical outcomes in the context of integrated bone transport techniques. A recent study reported that when using a non-eluting antibacterial-coated nail in cases of bone transport over nail (BTON), the EFT was reduced threefold compared to traditional external fixation methods, without increasing the complication rate (Corona et al., 2025).

### 3.3 Surgical technique

#### 3.3.1 Distraction histogenesis

Distraction histogenesis is a biologically friendly process that allows for the generation of high-quality biological tissue, including bone, that recapitulates the qualities of native tissue. The term “distraction histogenesis” is used specifically to indicate that all tissues in the extremity, including nerves, blood vessels, and other soft tissues, participate in the regenerative process. When applied specifically to bone, the term “DO” is used, and this is most often the greatest focus of attention. The key to this method lies in the biological principles governing DO (Huang et al., 2025). Following a low-energy osteotomy, the bone is then gradually distracted using a specific rate and rhythm, and continuous mechanical tension stimulates the periosteum and endosteum to produce new bone (Ilizarov, 1989). This process mimics natural bone growth during childhood, where bones lengthen through a similar mechanism.

For many years, this process began with the application of an external fixator, a circular frame composed of rings connected by adjustable rods and tensioned wires. This was then followed by a procedure where the involved bone is carefully broken or cut while preserving local soft tissue vascularity, creating a special type of osteotomy Ilizarov termed a “corticotomy”. This is essentially a low-energy osteotomy that is left undisturbed and anatomically reduced during a latency period of 5–7 d, to permit the fracture haematoma to consolidate and to allow the initial inflammatory response to resolve. Post-surgery, the external fixator is carefully manipulated to apply controlled forces across the corticotomy, and the distraction process commences. The hallmark of bone transport, as opposed to limb lengthening, is that the length of the limb in fact remains constant. The transport fragment is instead gradually translocated independently across the defect at a controlled rate of approximately 0.5–1 mm d^−1^, through evenly divided increments three or four times per day. This slow and steady movement is critical, as it allows the surrounding tissues, blood vessels, and nerves to adapt and also regenerate together with the bone.

Contemporary bone transport strategies can be broadly classified into three general categories: (1) powered by conventional external fixation alone (with or without an implant); (2) using external fixation augmented with cables to complete the transport; or (3) driven instead using powered telescopic intramedullary nails, eliminating the need for any external fixation. Nevertheless, the biological response to the mechanical conditions imposed are the same, regardless of which treatment strategy is employed and how the distraction process is ultimately completed.

#### 3.3.2 Corticotomy

Although segmental bone defects associated with FRI can be managed using either single-stage or multi-staged reconstructive strategies (depending on the extent of infection and soft-tissue involvement), the choice of corticotomy technique remains critical. In the context of FRI, selecting a low-energy corticotomy method that preserves local vascularity while minimizing additional tissue trauma is essential, as these biological factors directly influence regenerate quality and the success of bone transport in an infected environment. Currently, the fundamental principles of low-energy corticotomy are consistent across reconstruction methods. This includes the preservation of periosteal and endosteal vascularity through minimally traumatic percutaneous osteotomy techniques, although the execution varies depending on the chosen bone transport strategy. In classical circular external fixation, a low-energy corticotomy is typically performed percutaneously using multiple drill-hole perforations and then completed with an osteotome, thereby maintaining an intact periosteal sleeve and supporting reliable regenerate formation (Ilizarov, 1989; Paley and Tetsworth, 1991). This final step to complete the corticotomy is most often achieved via a closed rotational osteoclasis, and with an intact bone the distal segment of the limb can be easily manipulated. However, with a segmental defect, the presence of this discontinuity can create difficulties. A temporary half pin inserted into the eventual transport segment is a simple solution and acts as a means to clinically confirm that the corticotomy is complete. The Gigli saw can also be used in a percutaneous manner, with the advantage that a rotational osteoclasis is unnecessary (Paley and Tetsworth, 1991). In integrated fixation techniques, such as bone transport over a nail, the position and orientation of the corticotomy must be adapted to avoid contact with the intramedullary implant and to maintain alignment throughout distraction, whereas cable-based systems may require more proximal or distal corticotomy placement to optimize cable trajectory and reduce soft-tissue irritation (Bernstein et al., 2015; Quinnan and Lawrie, 2017; Seng and Oh, 2024). Fully internal methods employing motorized telescopic nails demand a highly precise corticotomy perpendicular to the nail axis, performed under fluoroscopic guidance to ensure smooth controlled transport of the segment (Gardner and Beason, 2021). In conjunction with a motorized telescopic nail, the percutaneous Gigli saw technique is a particularly elegant option. In that situation, the Gigli saw technique can be used to divide approximately 75 % of the bone's circumference, leaving one cortex intact initially. The nail can then be positioned definitively, including locking screws and blocking screws proximally and distally, before easily completing the corticotomy through the remaining intact cortex. This method minimizes potential angular deformities during transport by allowing the nail to be inserted and stabilized with the remaining anatomy undisturbed, before completing the corticotomy as the final step before wound closure. In selected situations, an oscillating saw with continuous saline irrigation may be used to create a controlled osteotomy; however, despite meticulous cooling, this technique carries a greater risk of thermal necrosis and partial disruption of the periosteal envelope, which may adversely affect regenerate quality. For this reason, saw-based osteotomies are generally reserved for anatomically restricted regions or when exact osteotomy orientation is required (for example, rotational deformity). Across all techniques, adherence to the biological principles described by Ilizarov remains essential to ensure predictable bone regeneration during distraction osteogenesis.

#### 3.3.3 Bone transport using external fixation

Traditionally, surgical methods utilize external fixators to support bone transport, and maintain alignment and stability. The predominant devices used for this purpose include classic ring and unilateral rail fixators. Various osteotomy approaches – including tandem, trifocal, tetra-focal, and penta-focal bone transport – have demonstrated success in accelerating the transport phase. Nevertheless, improvements in docking site healing and consolidation times remain minimal, with approximately one-third of patients requiring revision surgery due to docking site non-union (Liodakis et al., 2024).

**Figure 3:**
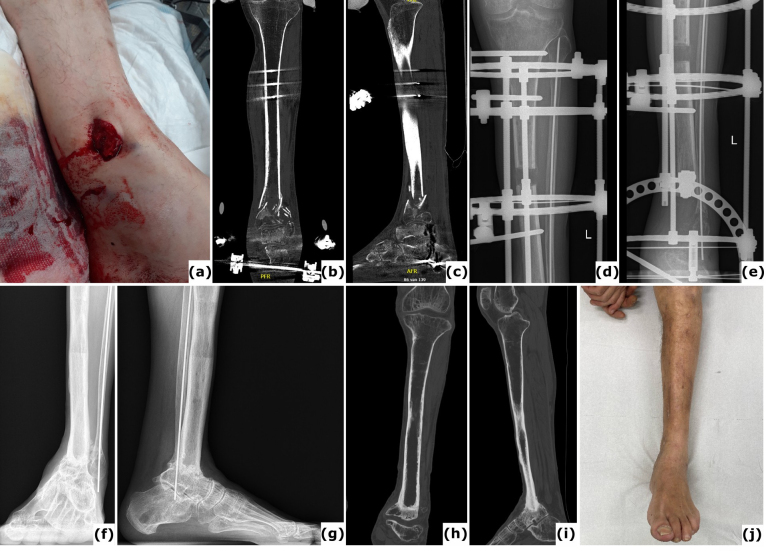
Bone transport utilizing external fixation. A 51-year-old patient presented with a Gustilo-Anderson type II open fracture of the distal tibia and fibula. **(a)** Clinical presentation upon admission to the emergency department, illustrating the open fracture. **(b–c)** Computed tomography (CT) images obtained at the time of referral, 3 months after initial stabilization with a standard external fixator, reveal absence of bone healing and significant destruction of the tibiotalar joint. **(d–e)** Anteroposterior radiographs demonstrate ongoing bone transport using a conventional ring fixator. Intraoperative cultures were negative. Note: Due to severe peripheral artery disease, there was a high risk of amputation; consequently, the corticotomy was performed at a lower level than the standard metaphyseal site. **(f–i)** CT scans, as well as anteroposterior and lateral radiographs, confirm complete consolidation at all sites at 2 years post-procedure. **(j)** Clinical evaluation at 2 years showed no signs of infection, and the patient was able to ambulate independently.

For defects measuring less than 8 cm, a bifocal approach is generally appropriate, involving a single osteotomy for transport (Fig. 3). Trifocal transport involves two osteotomies in motion, which may proceed in the same direction (tandem trifocal) or converge towards the centre of the limb. These procedures are complex, requiring thorough preoperative planning with calibrated imaging. Pin sites must be carefully positioned to avoid compromised tissues, and consideration of free flaps is essential. Determining the optimal timing for frame removal continues to be challenging, as sufficient healing must be achieved at both the docking site and within the distraction callus. Premature device removal can lead to complications such as fracture, secondary deformity, or non-union.

The technique of (circular) external fixation has been widely applied and the evolution of this technology has led to many new applications and designs, such as the hexapod external fixator (Sheridan et al., 2021). The hexapod frame consists of six telescopic struts connected to two full or partial rings via universal joints. The premise of this technology relies on projective geometry, where the adjustment of a single strut length will have an effect on the position of the two rings in space (Keshet and Eidelman, 2017). An internet-based software program is used to correct along six axes simultaneously (sagittal angulation and translation, coronal angulation and translation, and axial shortening and rotation). More recently, automated hexapod ring-fixation systems have been developed (Haruno et al., 2024). These systems eliminate the need for patients to manually adjust the struts, which may improve the treatment process and decrease the likelihood of complications associated with unintended adjustments.

**Figure 4:**
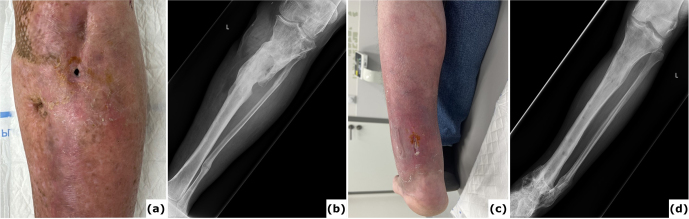
Chronic infection (osteomyelitis) following extended external fixation. **(a)** Clinical image presenting a fistula with purulent fluid drainage in a 64-year-old patient after multiple surgical procedures for fracture-related infection (FRI) of the proximal tibia with non-union. **(b)** Anteroposterior radiograph of the tibia showing consolidation of the proximal tibia fracture; the external fixation time (EFT) was 16 months. **(c)** Clinical image depicting a chronic wound on the posterior aspect of the ankle in a 54-year-old patient after several surgical interventions for FRI of the distal tibia. Bone transport was performed as treatment, with an EFT of 12 months. At referral, the patient had local and systemic signs of infection and was prescribed oral antibiotics. These episodes have recurred intermittently over 3 years following removal of the external fixator. **(d)** Anteroposterior radiograph of the tibia demonstrating consolidation at all sites.

Regardless of the specific technique employed, external fixation as a stand-alone method can present challenges for patients, given that bone regeneration may require as much as 2 months for every centimetre of bone transport. Furthermore, this approach is associated with several significant complications, including nerve damage, joint contracture, and scarring related to pin tracts. Pin site infections, in particular, merit special consideration. While these infections are often benign and typically manageable with appropriate care, the prolonged use of external fixation devices (12–18 months) in combination with recurrent pin site infections can increase the risk of developing chronic infection (osteomyelitis) (Frank et al., 2024) (Fig. 4).

#### 3.3.4 Bone transport using integrated fixation

Integrated fixation techniques have been developed to address the inherent limitations of solely external fixation methods by combining the advantages of both external fixators and internal implants (Bernstein et al., 2015). These integrated approaches were introduced as strategies specifically designed to facilitate the earlier removal of the external fixator following the transport phase, thereby significantly reducing the EFT. In addition, internal implants help to preserve bony alignment and stability, and also protect the distraction callus and docking site against fractures and deformities. The main difference between these integrated techniques is based on the timing of implant placement (Seng and Oh, 2024).

#### Intramedullary fixation after bone transport using external fixation

This technique involves transitioning from an external fixator to intramedullary nailing following a pin holiday, which is intended to reduce the risk of bacterial contamination and subsequent infection (Emara and Allam, 2008). However, this method may result in instability during the transition period, which can lead to fracture, spontaneous partial collapse of the distraction callus, malalignment, or non-union, and should therefore be used cautiously. Nevertheless, recent studies suggest that a pin holiday may no longer be necessary, given the implementation of antibacterial-coated nails as previously discussed (Corona et al., 2025; Pujol et al., 2023). 

#### Bone transport over a nail (BTON)

**Figure 5:**
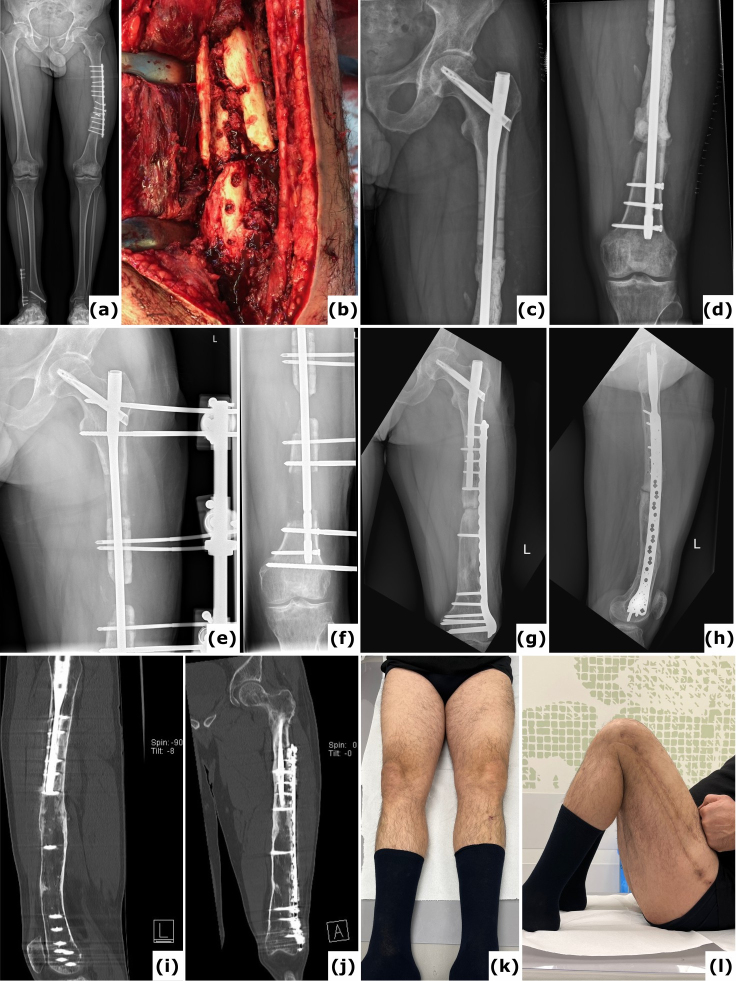
Trifocal transport over a nail. A 44-year-old patient presented with a fracture-related infection of the femur following plate osteosynthesis. **(a)** A long-leg radiograph obtained 6 months after the initial procedure at another institution shows an unhealed fracture and failure of the osteosynthetic construct. **(b)** An intraoperative clinical image displays the comminuted (avascular) fracture site. Cultures identified *Staphylococcus epidermidis*. **(c–d)** Anteroposterior radiographs of the left femur taken postoperatively reveal a large segmental defect, treated with an intramedullary nail and a PMMA spacer. **(e–f)** Anteroposterior radiographs document progression of the bone transport. **(g–h)** One-year anteroposterior and lateral radiographs demonstrate removal of the external fixator and revision to a nail/plate construct; cultures from this surgical intervention were negative. **(i–j)** Computed tomography (CT) images at 2 years confirm consolidation at all sites. **(k–l)** Clinical images at 2 years illustrate a full range of motion in both lower extremities.

Bone transport over a nail (BTON) is an advanced integrated fixation technique that utilizes an intramedullary nail to preserve alignment and provide stability across the segmental bone defect, whereas an external fixator facilitates the process of bone transport (Lu et al., 2022). By inserting the nail prior to commencing bone transport, the required ring or monolateral fixator construct is simplified to two segments rather than three, thereby reducing the number of trans-fixation pins or wires necessary (Fig. 5). This particular technique may necessitate nail modification, specifically by creating additional accessory holes adjacent to the docking site to secure the transported bone segment and to prevent recoil following the removal of the external frame. Such alterations can be achieved either by ordering custom-made nails or by adapting standard nails intraoperatively (Corona et al., 2025) (Fig. 2). The intramedullary nail ensures sustained stability throughout the consolidation phase, enabling earlier removal of the external fixator. However, BTON is associated with a potential risk of pin tract infections extending into the intramedullary canal. It is therefore essential to ensure that any pins or wires do not come into contact with the nail during the procedure, in order to minimize the likelihood of implant-related infection (Seng and Oh, 2024). To mitigate this risk, antibacterial-coated nails such as the ZNN™ Bactiguard^®^ nail have demonstrated promising outcomes (Corona et al., 2025).

#### Bone transport over a plate (BTOP)

Bone transport over a plate (BTOP) combines plate fixation with the use of an external fixator and is particularly appropriate for metaphyseal defects with limited bone stock, where nailing may be challenging. This technique is also applicable to bone defects in locations such as the forearm, which can present difficulties for intramedullary nailing and where ring fixators are sometimes poorly tolerated. A recent comparative study demonstrated that BTOP results in a significantly shorter EFT compared to BTON, with similar outcomes in segmental tibial bone defects (Park et al., 2021). The reduction in EFT was attributed to the stability provided by the plate, which supports both docking site union and consolidation of the distraction callus. In this series, however, the intramedullary nail was not modified to include locking holes adjacent to the docking site, preventing the transported segment from being captured by the nail. Consequently, the external fixator remained in place until both the regenerate and docking site healed, resulting in a longer EFT for the BTON group. Owing to its lower biomechanical stability compared to a nail, BTOP may necessitate supplementary plating when defects are situated near the metaphyseal-epiphyseal junction to ensure adequate stabilization during healing. Furthermore, it is important to closely assess the soft tissue status before selecting BTOP, as exposure and closure may present more challenges compared to BTON.

As mentioned earlier, both sequential and simultaneous integrated bone transport techniques can reduce the EFT. However, each method maintains an increased risk of infection due to possible contamination of the internal implant by the external fixation construct.

#### Balanced cable transport (BCT)

**Figure 6:**
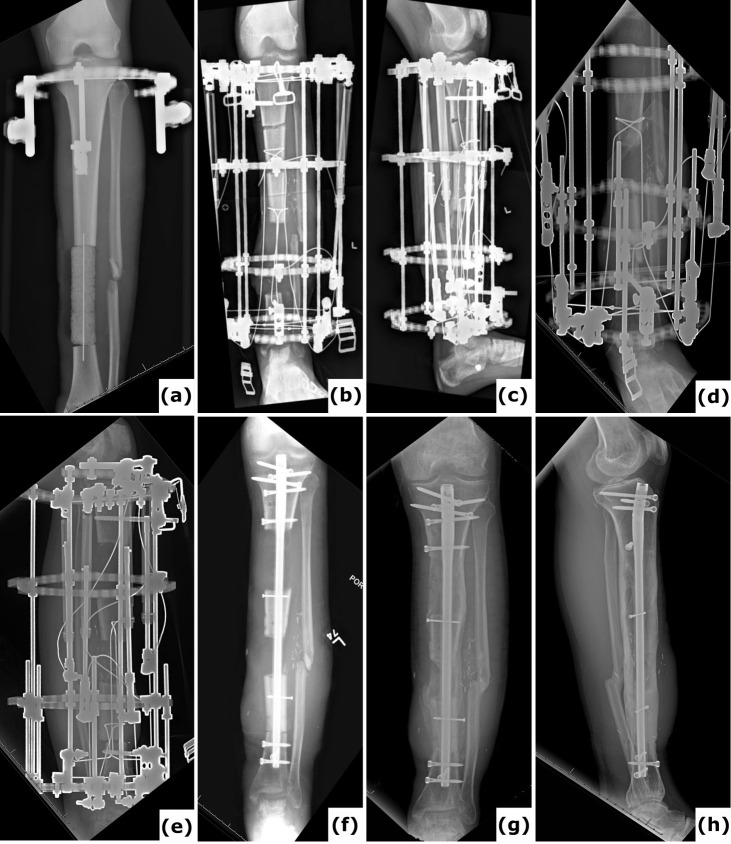
Trifocal balanced cable-transport and then nailing. **(a)** Anteroposterior radiograph of a 28-year-old patient with a Gustilo-Anderson type III open tibia and fibula fracture including 12.0 cm bone loss and 1.5 cm shortening from a motorcycle crash. During a first surgery, a temporary PMMA spacer was placed in the defect site and the bone ends were prepared for bone transport. **(b–c)** Anteroposterior and lateral radiographs after a tandem trifocal balanced cable transport external fixator were applied. Note there are two sets of cables and two distinct osteotomy sites for transport. **(d–e)** Anteroposterior radiograph with transport complete and additional lengthening at the proximal osteotomy site in process. **(f)** Anteroposterior radiograph after conversion to intramedullary fixation. The conversion was done shortly after the transport, and lengthening was completed. **(g)** Anteroposterior radiograph 7 months after the start of reconstruction, showing rapid progression of healing. **(h)** Lateral radiograph 2 years after the start of reconstruction, showing solid and mature healing at all sites.

Balanced cable transport (combined with circular external fixation) is a powerful integrated fixation technique. It can be combined with planned conversion to intramedullary fixation at the completion of transport (Balanced Cable-Transport And Then Nailing (BC-TATN)) (Quinnan and Lawrie, 2017), or the transport can be performed in an integrated fashion directly over a nail (Bernstein et al., 2018) (Fig. 6). Although technically complex, this technique substantially shortens the time of reconstruction, with fewer complications related to pin tracts. The advantage of the cable-pulley system is that the frame is used only in static mode; the cable that pulls the bone transport segment remains at the same exit point of the skin, thus limiting scarring. In addition, as soon as the distraction phase is completed, the external device can be removed. This substantially decreases the time that the external fixator needs to be in place. A clinical study on 14 tibia bone defects treated by a single surgeon reported excellent control of alignment, including the transport segment, easy conversion to allow limb lengthening, and full weight bearing throughout the treatment process (Quinnan and Lawrie, 2017). In addition, the method facilitates multifocal transport and safe conversion to intramedullary nail fixation, both of which can be used to substantially shorten the time of reconstruction. It has also been observed that the Bone Healing Index for the docking site and regenerate bone are significantly faster and more reliable when using BC-TATN compared to any other method. This is likely because of the stimulatory effect on healing of nailing through a fresh regenerate and the presence of the intramedullary contents delivered at the docking site, and is consistent with observations reported from Lengthening and Then Nailing (LATN) (Rozbruch et al., 2008).

#### 3.3.5 Bone transport using all internal fixation

#### Plate Assisted Bone Segment Transport (PABST)

Plate Assisted Bone Segment Transport (PABST) is a technique that completely eliminates the need for external fixation (Olesen et al., 2019). With this method, a locking plate spans the entire bone to maintain length and alignment while providing stability across the defect site. In the same construct, a motorized telescopic lengthening nail facilitates the movement of the transport segment of bone, either by lengthening or shortening the nail, until docking is achieved (Lance et al., 2024). Following docking, multiple management strategies may be considered, such as supplementary screw fixation utilizing the plate that has already been placed. A primary limitation of PABST is the extent of bone transport permitted, which is determined by the stroke length and design of the nail, and may necessitate further revision procedures for managing large defects. In addition, this technique requires intact metaphyseal-epiphyseal bone to anchor both the nail and the plate construct, making it most suitable for diaphyseal defects. As described for BTOP, it is important to carefully assess the soft tissue status as wound breakdown is a known complication (Gardner and Beason, 2021). Both techniques require a local soft-tissue envelope that is well perfused and compliant. When confronted with an adherent or fragile soft-tissue envelope, it may be necessary to employ additional flap coverage, and therefore combining these techniques might not be feasible under such conditions.

#### Bone transport nail

**Figure 7:**
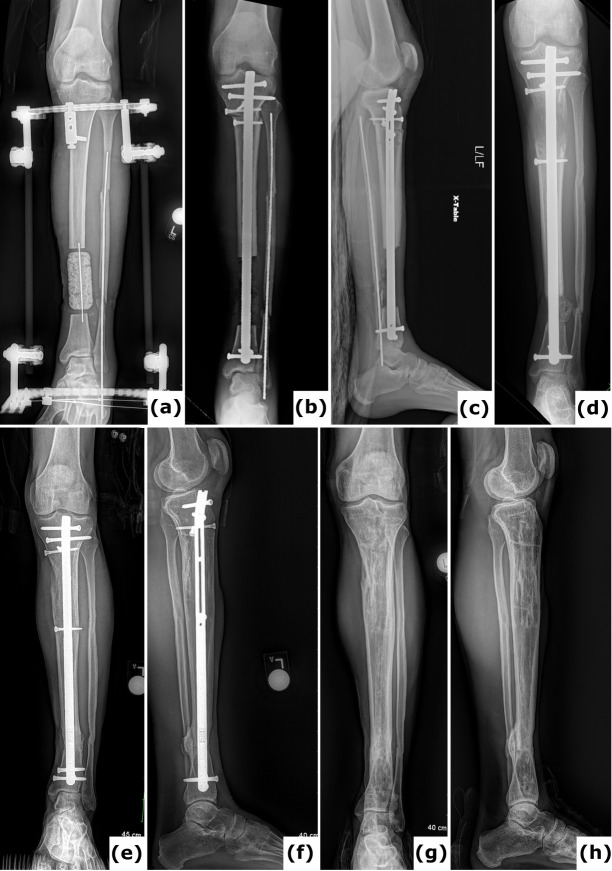
Bone transport nail. **(a)** Anteroposterior radiograph of a 26-year-old patient with a Gustilo-Anderson type III open tibia and fibula fracture, including 95 mm bone loss. In the initial surgery, a temporary PMMA spacer was placed at the defect site and the bone ends were prepared for bone transport. Provisional fixation was achieved using initial hybrid spanning fixation and an intramedullary wire in the fibula. **(b–c)** Anteroposterior and lateral radiographs display application of a bone transport nail with proximal and distal locking pegs, and required poller screw fixation to minimize drift during transport. **(d)** Anteroposterior radiograph demonstrates regenerate formation after a 2-week latency period and distraction at 0.6 mm d^−1^. **(e–f)** Anteroposterior and lateral radiographs document progressive healing subsequent to open docking. **(g–h)** Anteroposterior and lateral radiographs indicate solid and mature healing following removal of the bone transport nail 2 years post-injury.

This technique employs a specially designed motorized telescopic intramedullary device to span the entire bone and stabilize the defect (Fig. 7). The transport segment is captured by locking screws through a secondary central component of the nail, enabling bone transport through this mobile telescoping segment (Quinnan and Lawrie, 2017). Representing a relatively recent innovation, motorized nails do not provide the same strength for weight bearing as conventional trauma nails and are associated with substantially greater costs. Although this approach remains in the early stages of development, it has already seen substantial success; with continued experience and ongoing refinement of both the technique and technology, it is anticipated that all internal bone transport will continue to increase in popularity.

## 4 Discussion

The management of segmental bone loss remains one of the most complex challenges in orthopaedic reconstruction, especially when further complicated by FRI. Over the past century, various strategies have been developed to address critical-size bone loss (Bezstarosti et al., 2021). While numerous reconstructive strategies have been described for critical-sized bone defects, the success of any technique is ultimately determined by the adequacy of infection control. In clinical practice, failure to recognize or appropriately manage FRI remains a leading cause of reconstructive failure, prolonged morbidity, and, in some cases, limb loss.

Bone transport occupies a distinctive position among reconstructive options because it inherently aligns with the biological requirements of treating infected bone defects. By generating new bone through distraction osteogenesis, it avoids reliance on large structural grafts and minimizes the implantation of foreign material at the defect site during the early phases of reconstruction. For these reasons, bone transport has become a cornerstone technique for managing large defects in the context of FRI. However, the gradual nature of this process (in combination with external fixation) often resulted in prolonged courses of treatment that are difficult for patients to endure, sometimes requiring many months to complete the definitive reconstruction. This exposed these patients to substantial risks of complications including (pin site) infections, joint contractures, and debilitating pain, with a genuine impact on their quality of life while undergoing treatment (Abulaiti et al., 2017; Liu et al., 2023).

For the above-mentioned reasons, various technological innovations and surgical methods have been developed to shorten the period of external fixation. Systems using cables with a simplified external frame can be applied to assist in the transport process, followed by an early transition to intramedullary fixation, which substantially reduces the duration of external fixator use (Quinnan and Lawrie, 2017). Ongoing advancements in technology have facilitated a gradual transition from external fixation to the utilization of powered telescopic intramedullary nails for bone transport in appropriately selected patients in specialized clinical environments.

Importantly, these innovations do not replace the biological principles of distraction osteogenesis but rather optimize their clinical application. While integrated fixation techniques redistribute the risk profile of reconstruction – trading prolonged external fixation and pin-tract morbidity for implant-related considerations in a potentially contaminated environment – fully internal bone transport techniques substantially minimize the risk of infection by eliminating transcutaneous fixation altogether. In general, successful outcomes depend not solely on the chosen device but on a meticulous surgical technique, careful patient selection, and strict adherence to FRI management principles.

In summary, bone transport remains a fundamental treatment method for the management of large segmental bone defects, particularly when these are complicated by FRI. The efficacy of this technique relies on careful preoperative planning, advanced surgical expertise, and a thorough understanding of both mechanical and biological factors. Surgeons managing segmental bone defects must consistently consider the presence of FRI, even in the absence of overt clinical signs, and should adopt reconstructive strategies that prioritize infection eradication as the foundation for durable bone healing. Continued technological development combined with a sustained commitment to multidisciplinary care have fostered ongoing refinement of bone transport methods, resulting in better outcomes and renewed hope for patients facing some of the most demanding challenges in orthopaedic surgery. Effective treatment relies not only on sophisticated surgical interventions but also a holistic patient-centred strategy that comprehensively addresses the biological, mechanical, and infectious dimensions of the disorder. In this context, the participation of a multidisciplinary team is crucial, and management is preferably undertaken in dedicated specialist centres.

## 5 Conclusions

Bone transport is an advanced surgical technique that has made a significant impact in treating large segmental bone defects, especially when complicated by FRI. This technique utilizes controlled gradual mechanical distraction to promote bone regeneration according to the principles of DO, as introduced by Ilizarov. There are a number of different treatment strategies for bone transport, including traditional external fixation, integrated fixation techniques that combine external fixation with internal implants, and fully internal methods such as motorized telescopic intramedullary nails. Recent innovations, such as these motorized telescopic intramedullary nails, have led to a reduction or replacement of external fixation, making the procedure less burdensome for patients. The importance of considering FRI in every case is critical. Those surgeons with an interest in this challenging area of subspecialization continue to push the boundaries of limb salvage and reconstruction, often dealing with extremely demanding pathology. As the surgical skills, experience, and technology necessary to perform these procedures has progressed, the complexity of the cases treated has further expanded, and therefore a more sophisticated multidisciplinary approach in a specialized centre has become an essential component of contemporary clinical care.

## Data Availability

No data sets were used in this study.
